# Targeted location of microseismic events based on a 3D heterogeneous velocity model in underground mining

**DOI:** 10.1371/journal.pone.0212881

**Published:** 2019-02-25

**Authors:** Pingan Peng, Liguan Wang

**Affiliations:** School of Resources and Safety Engineering, Central South University, Changsha, Hunan, China; University of Science and Technology Beijing, CHINA

## Abstract

The accurate location of induced seismicity is a problem of major interest in the safety monitoring of underground mines. Complexities in the seismic velocity structure, particularly changes in velocity caused by the progression of mining excavations, can cause systematic event mislocations. To address this problem, we present a novel construction method for an arbitrary 3D velocity model and a targeted hypocenter determination method based on this velocity model in underground mining. The method constructs a velocity model from 3D geological objects that can accurately express the interfaces of geologic units. Based on this model, the block corresponding to the minimum difference between the observed arrival times and the theoretical arrival times computed by the Fast Marching Method is located. Finally, a relocation procedure is carried out within the targeted block by heuristic algorithms to improve the performance. The accuracy and efficiency of the proposed method are demonstrated by the source localization results of both synthetic data and on-site data from Dongguashan Copper Mine. The results show that our proposed method significantly improves the location accuracy compared with the widely used Simplex and Particle Swarm Optimization methods.

## Introduction

Microseismic monitoring is becoming a common tool with wide and successful applications in mining engineering [1]. It can provide important insight into a rock mass and quantify where a certain magnitude of induced rock fracturing is occurring within the volume [2]. Of the many processing procedures, event localization is crucial for the successful application of microseismic monitoring.

Since Geiger [3] proposed an iterative inversion method (known as Geiger’s method) for determining an earthquake’s epicenter in 1912, numerous approaches have been developed by researchers for automatic hypocenter location in the seismic and microseismic fields. These approaches can generally be classified into three broad categories: relative location methods, stack-based methods, and arrival time difference-based methods.

Relative location methods are multiple-event location techniques. The master-event (ME) method and the double-difference (DD) method are two representatives of this category. The ME method locates events relative to a previously located reference event (called the master event), the location of which is assumed to be accurate. This method assumes that the ray paths from the master and process events to the sensors have approximately the same take-off angles and traverse the same structures. Zhou et al. [4] made several improvements to the ME method and then applied it to relocate the Jiashi strong earthquake swarm in western China. In 2000, Waldhauser and Ellsworth [5] developed the double-difference (DD) location method, which is now widely used in earthquake location [6,7]. Recently, this method has been used to locate microseismic events induced by hydraulic fracturing. Chen et al. [8,9] incorporated back azimuth information based on the DD location method and obtained better relative event locations. Trugman [10] proposed a hierarchical clustering algorithm that can provide robust relocation results for earthquake sequences. Lin [11] presented a package called XCORLOC to improve relative earthquake location accuracy, which provides comparable results to the algorithms by [5] and [10]. Tan et al. [12] adopted the neighborhood algorithm and master station method for simultaneous microseismic velocity model inversion and source location. The relative location routine is helpful in cases in which the hypocentral separation between the events is much smaller than their distance to the sensors and the sizes of the inhomogeneities on the ray paths between the event and the sensors. This limitation restricts the application of relative location methods. In addition, these methods may suffer from a lack of events with known precise locations considering the complex conditions of deep subsurface engineering.

Stack-based methods are single event location techniques. One class of stack-based methods is based on the migration approaches used in seismic exploration. Gajewski and Tessmer [13] developed the time-reversal seismic event localization method, in which the reversely modeled wavefield focuses on the hypocenter of the seismic event. Nakata and Beroza [14] developed a new approach of time-reversal imaging by using the geometric mean as an imaging condition. Ding et al. [15] combined the reverse-time method with seismic ray tracing to avoid iterations and accelerate source localization algorithms. Another class of stack-based methods is based on the idea of delay and the sum of the traces. The Source Scanning Algorithm (SSA) [16] and the Kirchhoff reconstruction method [17] are applied to earthquake location. For microseismic applications, Grigoli et al. [18] presented a modified version of the SSA and demonstrated an application to mining-induced seismicity, and Gharti et al. [19] suggested a similar method. Using both rotation and stacking, the signal-to-noise ratio is enhanced, and the hypocenter is then located through a robust global search algorithm. All of these methods have the main advantages of requiring neither phase picking nor their identification. However, these methods are computationally intensive, so they are not suitable for real-time processing, and the energy focusing can be ambiguous for noisy data and very heterogeneous models [19].

The arrival time difference-based methods that are widely used in microseismic event location are single event location techniques. These methods focus on minimizing the residuals between the theoretical and observed arrival times of P- and/or S-waves. Classical solutions for solving the objective function include Geiger’s method [3], Newton’s method [20], and the Simplex method [21], which are integrated into many routine microseismic processing software packages. To be more robust, heuristic algorithms have been introduced in recent years, which are characterized by global optimization capabilities. Genetic algorithms were first presented by Sambridge [22] for earthquake hypocenter location and are extremely efficient compared with the classical solutions. Lagos and Velis [23] compared the microseismic event location results of the Very Fast Simulated Annealing (VFSA) and Particle Swarm Optimization (PSO) algorithms. These algorithms use a constant velocity of P- and/or S-waves, which leads to large location errors in heterogeneous environments. Considering different inhomogeneous models, variants of these methods have been presented for different applications. Zhou et al. [24] proposed an acoustic emission source location method for multilayered media that accounts for ray path refraction at the interfaces between layers. Feng et al. [25] proposed a sectional velocity model for microseismic source location in deep tunnels. Akram and Eaton [26] discussed various aspects of microseismic event locations using a 1D layered velocity model. Boltz et al. [27] discussed the influence of a mining-induced low-velocity zone (LVZ) on the locations of coal mining-induced seismicity and suggested that the location accuracy was greatly improved by considering the LVZ. These methods focus on specific velocity models or specific applications, which restrict the usage of these methods to more general applications.

Consequently, we focus on a more general and more accurate approach for microseismic location based on the arrival time difference in underground mining. As suggested by [28,29], all seismic event location techniques are sensitive to velocity model errors, even relative location techniques. Even though there are already some research papers concerning the heterogeneous velocity models for the accuracy of microseismic event locations, a general 3D velocity model construction method has not been presented. The large-scale mining excavation leads to various velocity zones, such as filled stopes, mined-out goafs, tunnels, and surrounding rocks. How to represent an accurate velocity model similar to the real world media, and how to locate the microseismic event based on this model, are two key factors to be addressed. In this paper, we propose a novel construction method for an arbitrary 3D velocity model and a targeted hypocenter determination method based on this velocity model in underground mining. Comparing with other studies, our work makes the construction of a complex 3D velocity model and the location of microseismic events based on the velocity model be possible. After the introduction, we present a general outline of the methodology and then provide a full description of the method. We then validate the method with two synthetic examples and a field application in underground mines.

## Methodology

### 3D velocity model construction

Grid-based models, in which each block has a velocity value, are typically used to represent the velocity structure in 3D cases. We can intuitively assign a velocity value to each block in 2D or, in some simple cases, in 3D. However, building a velocity model that accurately represents a real-world medium in 3D is complicated and tedious, especially when it is used to represent the surfaces of two different types of media. In 3D computer graphics, 3D modeling is widely used in a wide variety of fields, such as engineering, architecture, and movies. In recent decades, the mining companies have begun to construct 3D geological models as a standard practice. Currently, we can build 3D models using many kinds of open-source or commercial software, such as Surpac, Vulcan, Datamine, and Dimine. By developing a mathematical representation of the surface of an object in 3D, any heterogeneous geological medium can be easily represented. Therefore, any 3D velocity model can be easily constructed by converting 3D objects to grid-based velocity models. These 3D file formats are organized by layers. The objects within a layer can be defined as a velocity domain. Based on this idea, we present a novel construction method for arbitrary velocity models in 3D.

**Fig 1:**
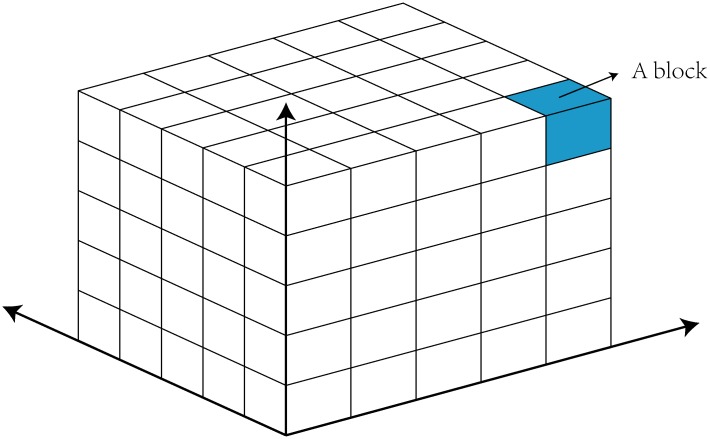
Schematic diagram of the 3D grid-based velocity model. Each block has a velocity value.

**Fig 2:**
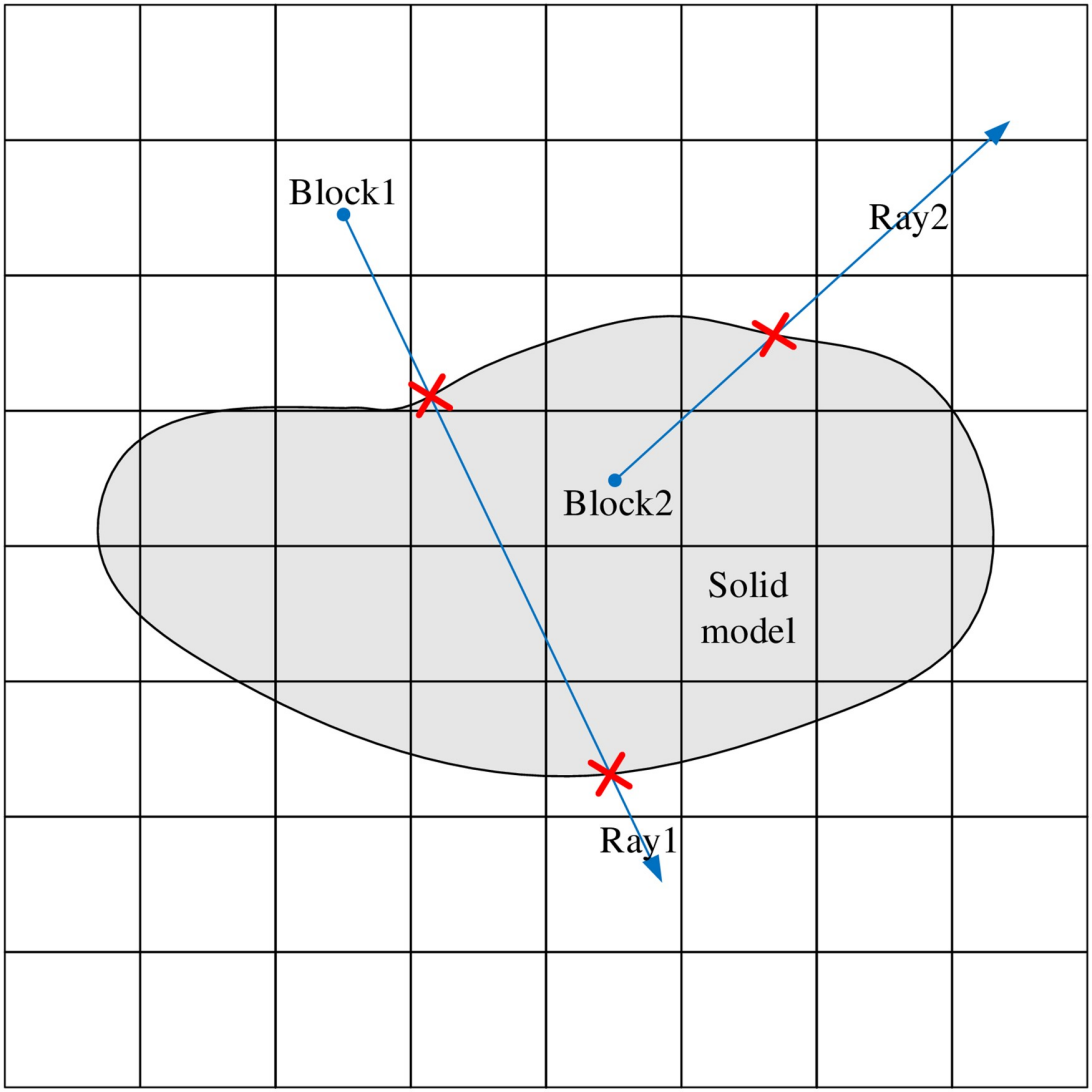
2D schematic demonstration of the velocity assignment method. Ray1 intersects the solid model twice, which means that Block1 is outside the solid model. Ray2 intersects the solid model once, which means that Block2 is inside the solid model.

For any geological solid model, we take its maximum outer contour as the size of the velocity model and then discretize the velocity model into blocks, as shown in Fig 1. Generally, the accuracy of travel time calculation will increase as the block size becomes smaller. However, it does not mean that we should make the block size as small as possible. First, when the block size is small to a certain extent, the improvement of accuracy is very limited. Second, the computational cost increase sharply as the block size gets smaller. Consequently, we should determine the block size by balancing the computational cost and the accuracy in practice. We then assign a velocity value to each block according to the property of the corresponding layer. The implementation details are as follows: shoot a ray from the centroid of a block and count the intersections with all of the polyhedrons. If the number is odd, the point is inside the polyhedron; if it is even, the point is outside. A 2D schematic diagram is shown in Fig 2. Finally, the velocity of the block is assigned based on the property of the polyhedron in which the centroid is located. The velocity is zero if no polyhedron encloses the block. The velocity model is built after all of the blocks have been assigned a velocity.

### 3D ray tracing

To model the traveltime from one grid to all grids at once, we select the Eikonal solvers for seismic ray tracing, which are more accurate than ray shooters and ray benders, especially in the presence of strong velocity gradients [30]. The Eikonal equation is a non-linear partial differential equation encountered in problems of wave propagation. It is derivable from Maxwell’s equations of electromagnetics and provides a link between physical (wave) optics and geometric (ray) optics. The Fast Marching Method (FMM) [31] is the most popular algorithm to solve the Eikonal equation.

Formally, the FMM was designed to solve nonlinear boundary value problems. Given a domain **X** and a velocity field function $F:\text{X}\to {\mathbb{R}}_{+}$ that represents the local speed of the motion, drive a system from a starting set **X**_s_ ⊂ **X** to a goal set **X**_g_ ⊂ **X** through the fastest possible path. For a general 3D grid, the Eikonal equation computes the minimum time-of-arrival function *T*(*x*) as follows:

$$\begin{array}{l} \left|\nabla T(x)\right|F(x)=1,\text{X}\subset {\mathbb{R}}^{N}\\ T(x)=0,x\subset {\text{X}}_{s} \end{array}$$ (1)

**Fig 3:**
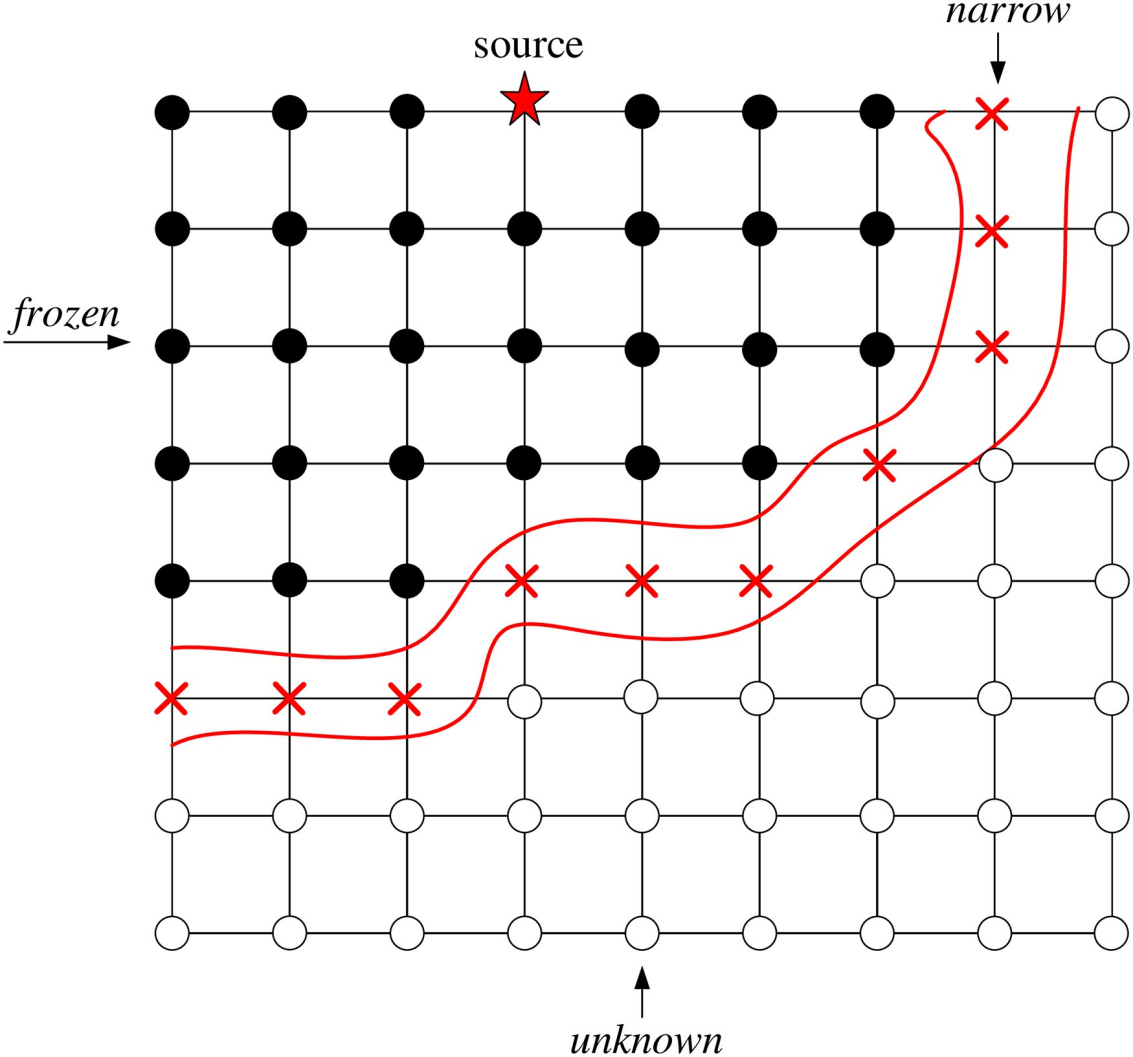
Schematic illustration of the fast marching method. See text for more details.

Eq (1) simply says that the gradient of the arrival time surface is inversely proportional to the speed of the front. According to Sethian [31], the grid points are divided into three classes, namely, *frozen*, points behind the wavefront and have been already computed; *narrow*, points on the wavefront awaiting assessment; *unknown*, which remains untouched ahead of the wavefront. In other words, the source positions at the beginning of the evaluation are considered *frozen*. Given that they are initial points, their traveltime is zero. All points that are one grid point away are taken as *narrow*, and their traveltime is computed analytically. All other grid points are marked as *unknown* and have an "infinitely large" traveltime value. These concepts are illustrated in Fig 3. The FMM algorithm can be summarized as follows.

1. LOOP: Among all *narrow* points, extract the point with minimum arrival time and change its tag to *frozen*.
2. Find its nearest neighbors that are either *unknown* or *narrow*.
3. Update their arrival times by solving Eq (1).
4. Go back to LOOP.

Once solved, *T*(*x*) represents a distance (time-of-arrival) field containing the time it takes to go from any point *x* to the closest point in **X**_s_ following the velocities on *F*(*x*). The domain is represented with a rectangular Cartesian grid $\text{X}\subset {\mathbb{R}}^{N}$, which contains the discretizations of the functions *F*(*x*) and *T*(*x*), **F** and **T**, respectively. For more detailed implementation of FMM in pseudo-code, please see S1 File. Ray paths are reversible, which means that the travel time tracing from the start to the goal is the same as that from the goal to the start. Therefore, we use receivers as starting points in practice, which will significantly reduce the computational cost. The travel times from a receiver to all of the grids are then computed by the FMM based on the constructed 3D velocity model, and the results are saved separately for further use.

### Block localization

Generally, the hypocenter is characterized by the minimum difference between the theoretical and observed arrival times. Thus, the objective function is expressed as follows:

$$f=\sum_{i=1}^{N}{\left|t_{obs}^{\left(i\right)}-t_{0}-t_{rt}^{\left(i\right)}\right|}^{m}$$ (2)

where *f* is the residual, *t*_obs_ is the observed arrival time, *t*_rt_ is the travel time computed by raytracing, *t*_0_ is the origin time of the source, *N* is the number of valid receivers, and *m* is the norm (*m*≥1).

The origin time is an unknown that cannot be ignored. Theoretically, the arrival time of a receiver minus the corresponding travel time along the path gives the origin time. Therefore, we can calculate the origin time by averaging the differences between the observed arrival times and the raytracing times of all receivers, which is expressed as follows:

$$t_{0}=\frac{\sum_{i=1}^{N}\left(t_{obs}^{\left(i\right)}-t_{rt}^{\left(i\right)}\right)}{N}$$ (3)

After loading the ray tracing results of all receivers, we calculate the value of Eq (2) from grid to grid. Finally, the block with the minimum objective function value is considered the preliminary location of the microseismic event. We refer to this block as the “targeted block”. The coordinate of the centroid of the targeted block is denoted as (*x*_*b*_, *y*_*b*_, *z*_*b*_).

### Targeted relocation

**Fig 4:**
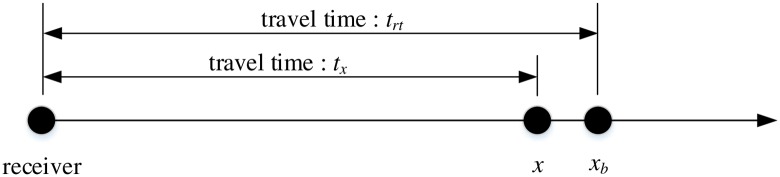
1D schematic illustration of updating theoretical travel time.

The precision of the preliminary location is controlled by the cell size. To improve the accuracy, a relocation procedure restricted within the targeted block is needed. Our goal is to find a constrained location that minimizes the differences between the actual travel times and the theoretical travel times through the media. Considering the velocity of the targeted block as a constant *v*_*b*_, let us denote (*x*, *y*, *z*) as the coordinates of the event location. For each receiver, the travel time is calculated by *t*_*obs*_*—t*_*0*_. The theoretical travel time will be updated according to the distance between the real location and the preliminary location. For simplicity, a 1D schematic illustration of updating theoretical travel time is shown in Fig 4. The Equation of updating theoretical travel time in 1D is expressed as follows:

$$t_{x}=t_{rt}-\frac{-\left(x-x_{b}\right)}{v_{b}}$$ (4)

In 3D cases, we introduce a gradient vector *p*, which indicates the component value of three axes toward the direction of the receiver. Thus, we present an objective function for the targeted relocation, which is expressed as follows:

$$\begin{array}{l} G=\sum_{i=1}^{N}\left|\left(t_{obs}^{\left(i\right)}-t_{0}\right)-\left(t_{rt}^{\left(i\right)}-\left[\frac{x-x_{b}}{v_{b}}\;\frac{y-y_{b}}{v_{b}}\;\frac{z-z_{b}}{v_{b}}\right]\left[\begin{array}{l} p_{x}^{\left(i\right)}\\ p_{y}^{\left(i\right)}\\ p_{z}^{\left(i\right)} \end{array}\right]\right)\right|\\ x,y,z\subset \psi \\ t_{0}\leq \text{min}(t_{obs}^{\left(\text{1}\right)},t_{obs}^{\left(\text{2}\right)},\cdots ,t_{obs}^{\left(N\right)}) \end{array}$$ (5)

where *G* is the objective function, *ψ* is the spatial domain of the targeted block, and (*p*_*x*_, *p*_*y*_, *p*_*z*_) are the gradients along the three axes. Denoting (*x*^(i)^, *y*^(i)^, *z*^(i)^) as the coordinates of the *i*-th receiver, the gradient vector is defined as follows for simplicity:

$$\begin{array}{l} p_{x}^{\left(i\right)}=h\left(\frac{x^{\left(i\right)}-x_{b}}{M}\right)\\ p_{y}^{\left(i\right)}=h\left(\frac{y^{\left(i\right)}-y_{b}}{M}\right)\\ p_{z}^{\left(i\right)}=h\left(\frac{z^{\left(i\right)}-z_{b}}{M}\right) \end{array}$$ (6)

where

$$\begin{array}{l} M=\text{max}(\left|x_{b}-x^{\left(i\right)}\right|,\left|y_{b}-y^{\left(i\right)}\right|,\left|z_{b}-z^{\left(i\right)}\right|)\\ h\left(j\right)=\left\{ \begin{array}{l} j,\;j=\pm 1\\ 0,\;-1<j<1 \end{array}\right. \end{array}$$ (7)

There are four unknowns in Eq (5), which is (*x*, *y*, *z*) and *t*_*0*_, and (*x*, *y*, *z*) are constrained within the targeted block. Therefore, we decided to use a global optimization algorithm and found the differential evolution (DE) approach [32] to be suitable for our purpose. The algorithm finds the parameter set minimizing the objective the function *G*. The outputs of DE are the targeted location and the origin time of the event.

## Synthetic tests

In this section, we conduct a series of synthetic tests to demonstrate the accuracy and efficiency of the proposed method.

### Example I

**Fig 5:**
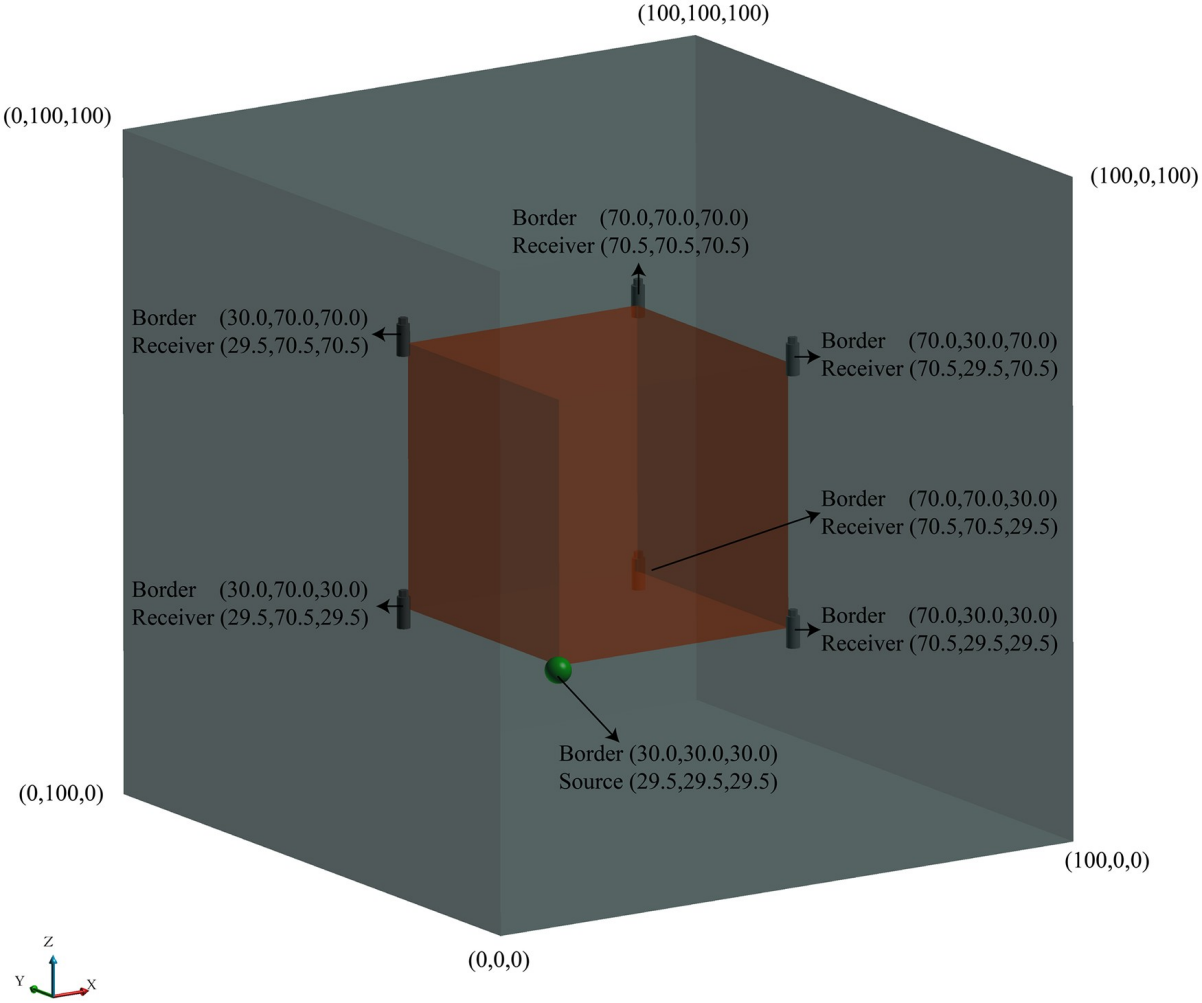
An idealized model. The model size is 100 m × 100 m × 100 m, and a void is located inside the cube. The border of the void is defined by the coordinates shown in the figure. The source is denoted by the green sphere, and the receivers are denoted by gray cylinders.

As shown in Fig 5, we build an idealized model with a size of 100 m × 100 m × 100 m. A void is located inside the cube and has the same centroid as the cube. The void’s size is 40 m × 40 m × 40 m. Six receivers are located on the corners of the void. The coordinates of the receivers and the vertex of the void are listed in Fig 5. The velocities of the cube and the void are assigned as 100 m/s and 0 m/s, respectively.

**Fig 6:**
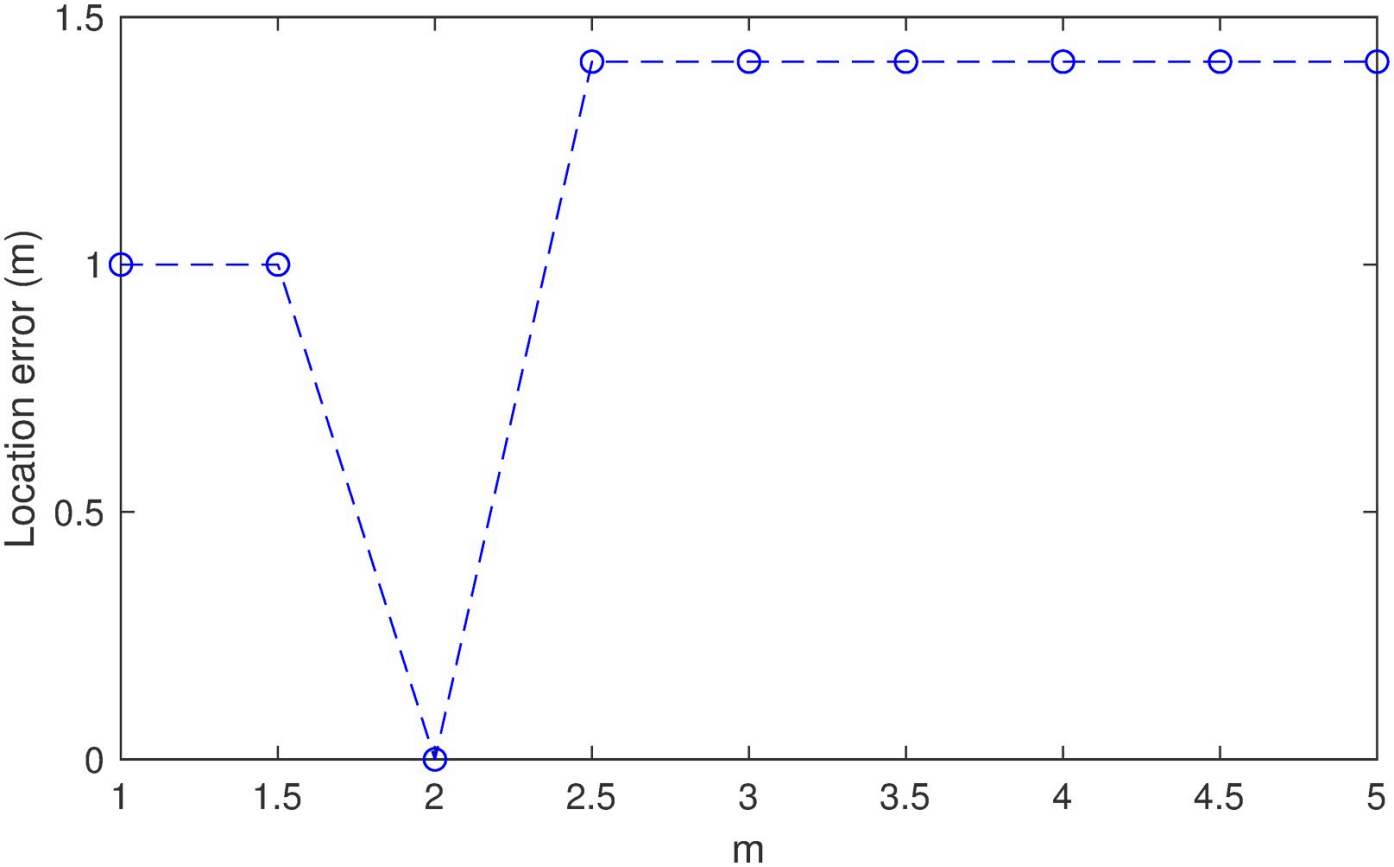
Location error versus the norm in example I.

The simulated source is located at (29.5, 29.5, 29.5). The relative origin time of this event is 100 ms. The path that the wavefront follows from the source to the receiver will be the shortest path. In this case, the traveltimes can be computed precisely by analytic geometry, as listed in Table 1. The traveltime plus the origin time gives the observed pick. In Eq (2), *m* is a parameter that will have an influence on the final result. In order to optimize the parameter, a comparison is conducted using a different norm to locate this synthetic event. The results are shown in Fig 6. It can be seen that the influence of *m* is very limited, but it locates the event precisely when *m* is 2. Thus, the optimized norm (*m* = 2) is used in the following synthetic tests and field applications.

**Table 1:** Input parameters for the event location in example I.

Receiver	x	y	z	Theoretical traveltime (ms)	Accurate picks (ms)	Noisy picks (ms)
S1	70.5	29.5	29.5	410.000	510.000	512.688
S2	70.5	70.5	29.5	579.828	679.828	688.997
S3	29.5	70.5	29.5	410.000	510.000	498.706
S4	70.5	29.5	70.5	579.828	679.828	684.139
S5	70.5	70.5	70.5	916.788	1016.788	1018.381
S6	29.5	70.5	70.5	579.828	679.828	673.290

Errors between the actual arrival and the picked arrival always exist. Therefore, for comparison, noisy picks are calculated by adding random perturbations to the accurate arrival times, as listed in Table 1. We then calculate the event locations for the two schemes using the proposed method as well as the widely used Simplex and PSO methods. Considering the constant velocity for Simplex and PSO methods is the same as the velocity of the cube, the results are shown in Table 2.

**Table 2:** Location results of the proposed method compared with Simplex and PSO in example I.

Method	using accurate picks				using noisy picks			
	x	y	z	Location error(m)	x	y	z	Location error(m)
Source	29.500	29.500	29.500	/	29.500	29.500	29.500	/
Simplex	39.143	39.032	3.931	28.9417	37.259	37.134	11.483	21.0498
PSO	20.470	20.470	18.208	17.0467	19.445	21.199	18.535	17.0365
Proposed method	29.500	29.500	29.500	0	28.500	29.500	29.500	1

Because the Simplex and PSO methods locate the event without considering the velocity inhomogeneity, the calculated coordinates are located far from the source with or without the picking noise. Using the accurate picks, the proposed method locates the source precisely. By adding picking noise, the location error of the proposed method is 1 m, which demonstrates the feasibility of the method.

### Example II

**Fig 7:**
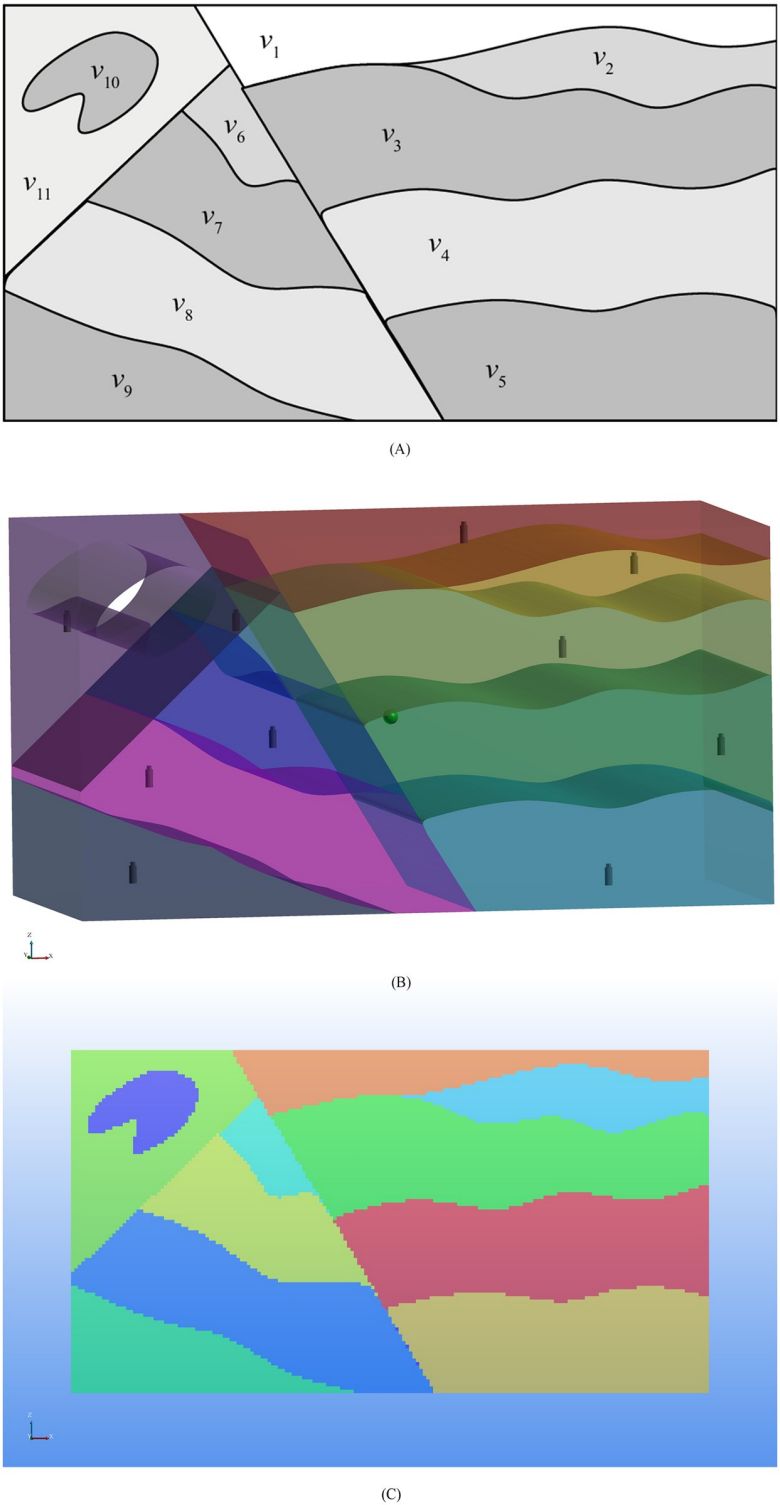
A complex synthetic velocity model. (A) The original 2D laterally heterogeneous velocity model presented by Rawlinson et al. (B) The 3D geological model constructed based on (A). (C) Front view of the 3D velocity model.

The velocity model presented above is a simple case that can be easily constructed without converting from 3D objects. However, it is difficult to build a velocity model that represents real media with realistic geological conditions. Therefore, in this section, a more complex 2D velocity model presented by Rawlinson et al. [33] is utilized. We extended the model from 2D to 3D. The original 2D model and the extended 3D geological model are shown in Fig 7(a) and 7(b), respectively. The size of the 3D model is 185 m × 100 m × 99 m. We randomly assigned the velocity values from *v*_1_ to *v*_11_ to be 900, 200, 500, 1000, 800, 300, 700, 100, 400, 0 and 600 m/s, respectively. We discretized the model using cells with a size of 1 m × 1 m × 1 m. The constructed velocity model is shown in Fig 7(c).

**Fig 8:**
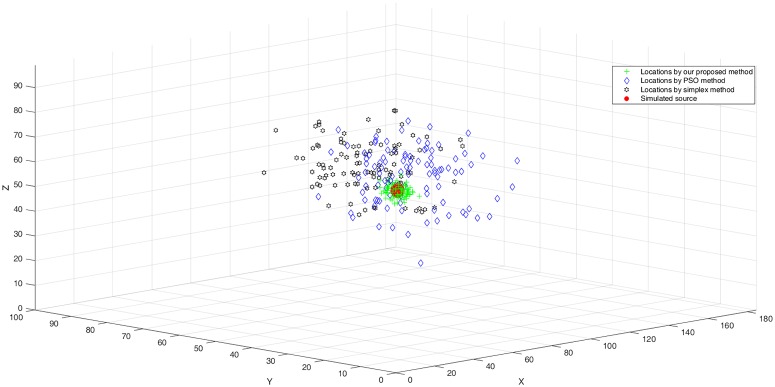
Location results of the proposed method compared with those of the Simplex and PSO methods in Example II.

Ten receivers are located in the ten velocity regions other than *v*_10_; their coordinates are listed in Table 3. The simulated event is located at the centroid of the model (92.5, 49.5, 48.5). We define observed picks by calculating the theoretical arrival times and adding normally distributed random perturbations. The mean of the normal distribution is chosen as 0, and the standard deviation is 10. This procedure is repeated 100 times. For repeatability, each simulation resets the seed value of the random number generator to ensure identical variations for each test depth. These observed picks are then used as inputs to the proposed method as well as to the Simplex and PSO methods to evaluate how well they perform in determining the synthetic event’s location. As shown in Fig 8, the locations calculated using the proposed method are very close to the source. However, the locations calculated using the Simplex and PSO methods are scattered at greater distances around the source, which demonstrates the accuracy of the proposed method in complex structures.

**Table 3:** Receiver coordinates.

Receiver	x	y	z
S1	112.806	50	96.306
S2	163.985	80	84.847
S3	133.402	20	67.556
S4	179.338	40	39.254
S5	145.062	20	7.004
S6	55.114	70	73.072
S7	60.918	50	43.369
S8	23.947	30	35.189
S9	15.562	10	11.337
S11	13.55	90	72.455

## Field application

**Fig 9:**
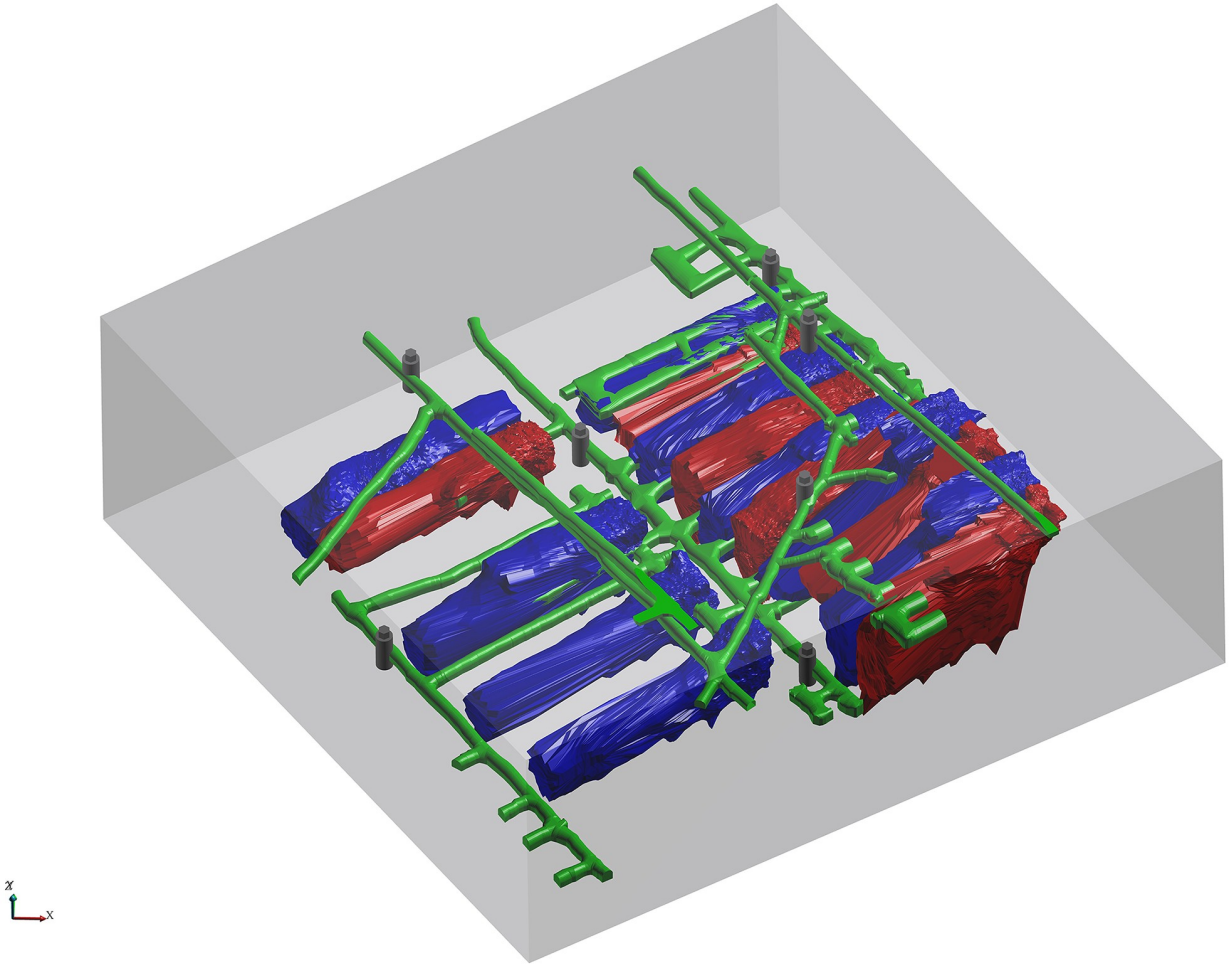
3D model of the microseismic monitoring area in the Dongguashan Copper Mine. The model is filled with the surrounding rocks. The mined-out goafs are represented by a blue color. The filled stopes are represented by a red color. The tunnels are represented by a green color. The receivers are represented by a black color.

The Dongguashan Copper Mine is located in Tongling, Anhui Province, China. Because it is a deep underground mine, Dongguashan suffers from rockburst hazards that can result in casualties and damage to equipment. Therefore, a microseismic monitoring system was installed in Dongguashan with 7 accelerators embedded at the -730 m level and the -790 m level. The coordinates of these receivers are listed in Table 4, and the layout is shown in Fig 9. Microseismic events were processed using commercial software by analysts in daily monitoring. The constant velocity used in processing is 5730 m/s, which is calibrated by blasts with known coordinates. The distance from the blast to each of the sensors is calculated and plotted against the absolute or relative arrival time recorded on the seismic system. Then the velocity is simply the slope of the best-fit line. According to the report by Dongguashan Copper Mine, location accuracy by traditional methods turned out to be poor. As we known, locating induced seismicity in active underground mines is a challenging problem because of the presence of complex structures, such as mined-out goafs, filled stopes and rock strata. As demonstrated above, we can construct an arbitrary 3D velocity model and accurately locate events using this model with our method. By considering the velocity inhomogeneity, the proposed method provides better location quality than traditional methods. To validate this, controlled blast experiments with small amounts of explosives were carried out in five different locations in the monitoring area.

**Table 4:** Coordinates of the accelerators installed in Dongguashan Copper Mine.

Receiver	x	y	z
R1	3909.1015	2278.5813	-770.4217
R2	3973.3256	2376.3145	-770.3028
R3	3915.6763	2376.2952	-735.163
R4	4054.2134	2269.313	-765.8802
R5	4035.9158	2459.6414	-769.6717
R6	4051.6684	2384.7272	-721.4167
R7	4052.434	2313.5298	-727.2345

**Fig 10:**
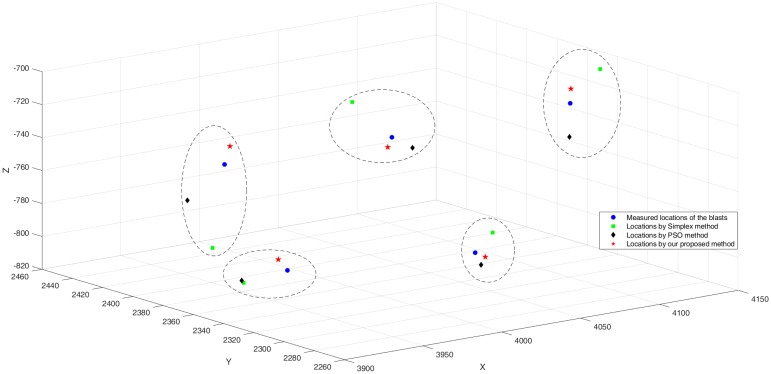
The location results for controlled blast experiments in five different locations. The actual locations of the blasts are marked with blue circles. Locations by Simplex, PSO and our proposed method are marked with green squares, black diamonds and red pentagrams.

Based on the survey data provided by Dongguashan, the filled stopes, mined-out goafs, tunnels, and surrounding rocks within the monitoring area were defined in the 3D modeling software, as shown in Fig 9. The P-wave velocities of the filled stopes and surrounding rocks were taken as the mean values of laboratory tests, and the P-wave velocities of the mined-out goafs and tunnels are similar to the wave velocity of air (Table 5). Five blasts were recorded by the microseismic monitoring system, and the observed P-wave arrivals were picked by analysts. The coordinates of the blasts were pre-measured and shown in Table 6. The locations of the blasts were then calculated using our method as well as the Simplex and PSO methods, and the results are shown in Fig 10 and also listed in Table 6. Fig 10 shows that the located events of the proposed method are more accurate than the results of the Simplex and PSO methods, which is much closer to the actual blast location in terms of distance. It can be seen from Table 6 that the mean absolute location error using the proposed method is 7.651 m, which is better than those of the Simplex method (31.305 m) and the PSO method (23.022 m).

**Table 5:** P-wave velocities of the Mine Model.

Name	Surrounding rocks (m/s)	Filled stopes (m/s)	Mined-out goafs (m/s)	Tunnels (m/s)
Velocity	5500	1900	340	340

**Table 6:** Location results of the pre-measured blasts in the Dongguashan Copper Mine.

Blast	Source			Simplex				PSO				Proposed method			
	x	y	z	x	y	z	Location error(m)	x	y	z	Location error(m)	x	y	z	Location error(m)
B1	3931.947	2330.966	-790.571	3921.485	2349.121	-801.370	23.573	3919.966	2348.618	-799.479	23.119	3929.352	2334.293	-784.440	7.443
B2	3990.333	2267.695	-772.276	4012.250	2278.967	-766.904	25.224	4002.686	2276.633	-784.208	19.361	3993.634	2264.427	-774.502	5.151
B3	4005.131	2338.181	-724.124	3985.471	2343.918	-701.082	30.828	4023.934	2344.165	-735.342	22.698	4001.946	2337.603	-729.458	6.239
B4	3990.380	2433.451	-764.304	3971.203	2421.308	-808.46	49.648	3979.001	2446.152	-787.739	28.982	3994.637	2434.325	-754.214	10.986
B5	4125.816	2346.109	-726.016	4147.391	2348.710	-709.572	27.252	4116.489	2336.925	-742.373	20.949	4128.321	2348.275	-718.259	8.434
Mean	/	/	/	/	/	/	31.305	/	/	/	23.022	/	/	/	7.651

## Conclusion

We present a novel construction method for an arbitrary 3D velocity model and a targeted hypocenter determination method based on this velocity model in underground mining. The method constructs the velocity model by converting 3D geological objects that accurately express the interfaces of realistic geology. Based on this model, the block corresponding to the minimum difference between the observed arrival times and the theoretical arrival times computed by the FMM method is located. Finally, a relocation procedure is carried out within the targeted block to improve the location accuracy. We successfully tested this approach with both synthetic and field-data applications in underground mining. The results show that the methodology can greatly improve the location accuracy compared to the Simplex and PSO methods in heterogeneous media. It should be noted that accurate velocity structures are a prerequisite for reducing the location errors and need to be obtained through other geophysical methods, which is not a focus of this paper.

## Supporting information

S1 File Implementation of FMM. (PDF)
