# Supplementary material for: Targeted location of microseismic events based on a 3D heterogeneous velocity model in underground mining
Source: PLoS One. 2019 Feb 25;14(2):e0212881. doi: 10.1371/journal.pone.0212881 (PMC6388919; doi:10.1371/journal.pone.0212881)
Supplement: S1 File — (PDF) [file pone.0212881.s001.pdf]

Implementation of FMM

Let the grid point x_i represents the point in the space corresponding to index i of the grid. For simplicity of notation, $T_\mathrm{i} = T(x_i)$; that is, T_i represents an approximation of the real value of the function $T(x_i)$. The set of Von-Neumann neighbors of grid point x_j is denoted as $\mathcal{N}(x_j)$. The pseudo-code of the FMM is detailed as follows.

Procedure FMM($\mathcal{X},\mathcal{T},\mathcal{F},\mathcal{X}_s$)

Initialization:

$Unknown \leftarrow \mathcal{X},\ \ Narrow \leftarrow \varnothing,\ \ Frozen \leftarrow \varnothing$

$T_\mathrm{i} \leftarrow \infty \forall x_i \in \mathcal{X}$

for $x_i \in \mathcal{X}_s$ do

$T_\mathrm{i} \leftarrow 0$

$Unknown \leftarrow Unknown \setminus \{x_i\}$

$Narrow \leftarrow Narrow \cup \{x_i\}$

Propagation:

while $Narrow \neq \varnothing$ do

$x_{min} \leftarrow arg\min_{x_i \in Narrow} \{T_i\}$

for $x_i \in \left(\mathcal{N}(x_{\min}) \cap \mathcal{X} \setminus Frozen\right)$ do

$\tilde{T}_i \leftarrow SolveEikonal(x_i, \mathcal{T}, \mathcal{F})$

if $\tilde{T}_i < T_i$ then

$T_i \leftarrow \tilde{T}_i$

if $x_i \in Unknown$ then

$Narrow \leftarrow Narrow \cup \{x_i\}$

$Unknown \leftarrow Unknown \setminus \{x_i\}$

$Narrow \leftarrow Narrow \setminus \{x_{\min}\}$

$Frozen \leftarrow Frozen \cup \{x_{\min}\}$

return $\mathcal{T}$
